# Association between radicular low back pain and constipation: a retrospective cohort study using a real-world national database

**DOI:** 10.1097/PR9.0000000000000954

**Published:** 2021-08-26

**Authors:** Robert James Trager, Shaffer R.S. Mok, Kayla J. Schlick, Jaime A. Perez, Jeffery A. Dusek

**Affiliations:** a Connor Integrative Health Network, University Hospitals Cleveland Medical Center, Cleveland, OH, USA; b UH Digestive Health Institute, University Hospitals Cleveland Medical Center, Cleveland, OH, USA; c Clinical Research Center, University Hospitals Cleveland Medical Center, Cleveland, OH, USA

**Keywords:** Low back pain, Constipation, Radiculopathy, Electronic medical records, Confounding variables, Propensity score

## Abstract

**Introduction:**

Previous observational studies have reported an association between lumbosacral radiculopathy (LSR), a form of low back pain (LBP) with nerve root involvement, and constipation. However, it is unclear whether this association is due to confounding variables such as comorbidities and medications.

**Objectives:**

This study explores the possible association between LSR and constipation, with the hypothesis that adults with LSR have increased odds of developing constipation compared with those with nonradicular LBP.

**Methods:**

Adults aged 18 to 49 years with incident LSR and nonradicular LBP were identified from a national 70 million patient electronic health records network (TriNetX). Propensity score matching (PSM) was used to control for covariates and determine the odds ratio (OR) of constipation over a 1-year follow-up. Lumbar stenosis, cauda equina syndrome, and inflammatory bowel diseases were excluded.

**Results:**

After PSM, 503,062 patients were in each cohort. Before PSM, the likelihood of constipation was identical between cohorts (LSR 10.8% vs 10.9%; OR [confidence interval] = 0.99 [0.98-1.0], *P* = 0.251). This association was unchanged after PSM (LSR 10.8% vs 11.1%; OR [confidence interval] = 0.98 [0.97-0.99]; *P* = 0.003).

**Conclusions:**

The study hypothesis can be refuted given that the OR approximated the null in a large propensity-matched sample. Patients with LSR have equivalent odds of constipation compared with those with nonradicular LBP, suggesting that LSR is not a direct cause of constipation. The similar risk of constipation between cohorts could be explained by factors common to LBP in general, such as pain severity, physical inactivity, and constipating medications.

## Abstract 2

Supplemental Digital Content is Available in the Text.

The results from a large sample suggest there is no clinically significant increase in odds of constipation in lumbosacral radiculopathy compared with nonradicular low back pain.

## 1. Background

Lumbosacral radiculopathy (LSR) is a subset of low back pain^37^ (LBP) affecting one or more lumbar or sacral nerve roots that can involve radiating pain into the lower extremity, weakness, and/or sensory loss.^41^ Lumbosacral radiculopathy is pathoanatomically and clinically distinct from nonradicular LBP, which has different clinical features^8^ and treatment.^25^ However, LSR is less common than nonradicular LBP, having a point prevalence of 2% to 13%.^18^ Degenerative conditions of the low back such as lumbar disk herniation (LDH) and lumbar spinal stenosis (LSS) are the most common causes of LSR, although rarely LSR results from nondegenerative conditions such as infections or neoplasms.^41^

Constipation is a common symptom that affects 3% to 27% of the adult population^28^ and often involves neurological mechanisms.^26^ It is defined as a change in bowel habits involving a reduced frequency of defecation or change in stool caliber to hard or dry feces. Subcategories of constipation include slow transit, in which transit time is increased; defecatory disorders, which involve a decreased ability to evacuate stool from the anorectum; and constipation-predominant irritable bowel syndrome (IBS), which involves pain associated with altered bowel habits.^26^

There is neuroanatomical evidence that the large intestine receives innervation from the lumbosacral nerve roots. However, colonic motility is self-regulated by the enteric nervous system, and it is unclear to what extent this lumbosacral innervation influences motility. The proximal colon is innervated by lumbar splanchnic nerves while the distal colon is innervated by lumbar splanchnics and sacral pelvic nerves.^4^ The cell bodies of these nerves are located within the lumbosacral dorsal root ganglia.^4^ These afferent nerves enable the perception of colonic fullness^4^ and are believed to mediate colonic motility through a spinal defecatory reflex that engages the parasympathetic nervous system.^34^

Owing to the close neuroanatomical relationship between the colon and lumbosacral nerve roots, we suspected that a neurological lesion of these nerves (ie, LSR) could reduce colonic motility. Our hypothesis is based on the possibility for LSR to (1) interrupt the spinal defecatory reflex, leading to decreased colonic parasympathetic activity and motility and (2) reduce afferent signaling from the colon, leading to reduced colonic sensation and a corresponding decreased urge to evacuate.

The ancient physician Hippocrates may have been the first to propose an association between LSR and constipation. He noted that patients with sciatica who required medicine to move their bowels would have longer-lasting symptoms.^13^ He and other ancient physicians recommended laxatives for these patients.^23^ Despite these historical anecdotes, there has been limited research exploring the association between LSR and constipation.

One small study of treatments for radicular sciatica reported that 45% of patients were constipated at baseline.^1^ Another study documented a high incidence of constipation in patients after thoracolumbar fusion surgery.^36^ A study documenting the disability level of patients with LSS noted that constipation was a common complaint, but this finding was not further investigated.^50^ However, because these studies have a small sample size and did not control for confounders, it is not possible to make firm conclusions about the etiology of constipation in these patients.

One major confounder in this area of research is that medications commonly used for LSR increase the risk of constipation. An example of this is from a study that found acetaminophen and nonsteroidal anti-inflammatory drugs (NSAIDs) had significant associations with constipation.^5^ Opioids are a well-known risk for constipation, with 40% to 95% of patients developing this side effect.^22^ There is some evidence that other medications for LSR^7^ including benzodiazepines^3,32^ and pregabalin^15^ also increase the risk of constipation.

Bowel or bladder dysfunction has been better described in patients with cauda equina syndrome (CES), a more severe type of lumbosacral nerve root injury compared with LSR.^40,41^ Constipation has been reported in patients recovering from surgery for discogenic CES^29,40^ and in those developing CES as a surgical complication.^24^This study sought to determine whether patients with LSR have greater odds of developing constipation compared with those with nonradicular LBP, before and after controlling for covariates using propensity score matching (PSM). We hypothesize that adults with LSR have greater odds of constipation than those with nonradicular LBP. To the best of our knowledge, no previous study has investigated an association between LSR and constipation using a large database of patients while controlling for confounding variables.

## 2. Materials and methods

### 2.1. Study design

This study followed a preregistered protocol^44^ available at osf.io/np3cj and is reported according to the STROBE statement.^48^ The original protocol was modified to improve the specificity of the LSR cohort by excluding patients with LSS (by code and age restriction), omitting the ICD-9 inclusion codes for radiculitis of “unspecified” regions of the spine (ie, 729.2 and 724.4), and reduce confounding by propensity matching for IBS. This study incorporates a new-user, active-comparator, retrospective observational design using aggregated, multisite, EHR-based real-world data. The patient population includes adults from age 18 to 49 years of any sex.

### 2.2. Setting and data source

This study used a subset of the TriNetX (TriNetX, Inc., Cambridge, MA) research network, which has been described previously.^43^ This is a federated national network that includes aggregated, deidentified data from over 70 million patients across EHRs of 51 health care organizations (HCOs). Information relating to participating HCOs is kept anonymous by TriNetX; therefore, geographic or institutional information are unavailable. However, a typical participating HCO represents a large academic health center with inpatient, outpatient, and specialty care services. To safeguard protected health information, TriNetX deidentifies data by restricting the population to patients younger than 90 years in accordance with the Health Insurance Portability and Accountability Act, which considers age >90 years as an identifier. In addition, queries that return patient counts <10 are rounded up to 10. These safeguards were not pertinent to our study which focused on patients <50 years and those with constipation which is a common outcome. TriNetX enables queries using standardized terminologies such as *International Classification of Disease* (ICD) and *Current Procedural Terminology* codes.

At University Hospitals of Cleveland, access to TriNetX is managed by the University Hospitals Clinical Research Center. A TriNetX search query was performed on June 16, 2021, to identify patients admitted between January 1, 2004, and June 16, 2020. Studies of this type are determined by the University Hospitals Institutional Review Board to be exempt from requiring informed consent. An individual level data set was not needed because of the study aims and required statistical analyses. This type of data set is necessary when the statistical techniques to be used exceed the capabilities of the analytical tools available within TriNetX. For this study, the statistical tools within the TriNetX platform were sufficient to compare baseline characteristics and perform PSM, and individual patient data that would be needed to conduct additional testing such as a multivariate logistic regression for an outcome were not necessary.

### 2.3. Participants

A new-user, active-comparator design was used in this study to reduce prevalent user bias and increase the comparability of cohorts.^12^ Because TriNetX identifies patients with the first occurrence of a given diagnosis by default, our study included patients with new-onset (incident) LBP. The new-user design was applied to the outcome of interest by excluding patients with a history of constipation (Supplementary material, Figure 1, available at http://links.lww.com/PR9/A128). An active comparator of LBP was used instead of healthy controls because these patients have a related health care problem causing them to seek care, which allowed for greater comparability to the LSR cohort.

### 2.4. Eligibility criteria

Adult patients were included who had incident, nonurgent, radicular, or nonradicular LBP and no previous history of constipation. An age range of 18 to 49 years was used to make the LSR cohort more specific to radiculopathy resulting from LDH rather than LSS. Previous studies have shown that LSR related to LDH is more common in patients in their 30s and 40s,^14,52^ and becomes less common after age 50, during which point LSS becomes more common.^14^ A cutoff point of 50 years has been used previously to help distinguish between patients with LDH and LSS in claims data.^11^

Specific exclusions for both cohorts were used to make the query more specific: (1) neurological disorders at an alternate anatomic site that could cause LBP, (2) serious, urgent, or emergent low back–related pathology, and (3) inflammatory bowel disease (Supplementary material, Table 1, available at http://links.lww.com/PR9/A128). Severe low back–related pathology such as CES, neoplasms, infection, and fractures were excluded by using ICD-9 codes that were used in previous studies for this purpose.^6,10,17^ We chose not to exclude an exhaustive list of neurological disorders because doing so could unintentionally exclude patients with LSR and because certain disorders could be controlled for later using PSM.

Diagnostic codes corresponding to LSR and nonradicular LBP were adapted from previous studies.^6,10,17,47^ Definitions of codes were identified using a website and converted between ICD-10 and ICD-9 by this site's online conversion tool.^42^ Our LSR cohort was intended to be specific by only including codes that indicate nerve root involvement related to the low back, particularly those associated with discogenic radiculopathy, the most common cause of LSR.^19^

Certain ICD codes are neutral with regards to the presence of nerve root involvement, such as those for LDH or spondylolisthesis, which can cause LBP with or without LSR.^39^ To account for this ambiguity, this study did not include patients with these diagnoses unless they had a specific LSR diagnosis (Supplementary material, Table 2, available at http://links.lww.com/PR9/A128).

The nonradicular LBP cohort included patients with the code for “low back pain” (ICD-9: 724.4). Diagnoses specific to LSR as well as those that could relate to LSR, such as sciatica, and disk displacement (Supplementary material, Table 3, available at http://links.lww.com/PR9/A128) were excluded from the nonradicular LBP cohort, in addition to the exclusions for both cohorts (Supplementary material, Table 1, available at http://links.lww.com/PR9/A128). Spondylolisthesis and spondylolysis were also excluded from the nonradicular LBP cohort as some research has found an association between these conditions and LSR.^27,39^

### 2.5. Variables

For the purposes of this study, constipation was defined using a composite outcome of constipation diagnoses and laxative medications (Supplementary material, Table 4, available at http://links.lww.com/PR9/A128) over a 1-year follow-up window. This phenotype was developed using a previous study as a starting point^45^; however, our study differs by not using the code for IBS as an outcome. The IBS code was removed for our study because the IBS patterns of “mixed diarrhea and constipation” or “diarrhea only” are more common than “constipation only.^31^” Although ICD-10 provides more detailed codes specifying the presence or absence of constipation and diarrhea associated with IBS, general equivalence mapping is performed automatically in the TriNetX platform.

Our study included patients taking laxatives in the constipation outcome by using the drug class GA200 in the Veterans Health Administration National Drug File (VANDF).^20^ This strategy was intended to increase the sensitivity for detecting patients with constipation, considering some patients using laxatives may not have been diagnosed as such in the EMR, as a previous study found that many patients do not discuss constipation with their medical provider.^46^ Another related benefit is that this strategy may identify patients taking laxatives for constipation related to a “constipation only” form of IBS, which was not included in the constipation outcome.

### 2.6. Potential confounders

This study used PSM, a method of balancing covariates between cohorts recommended to reduce bias in retrospective observational studies.^12^ Covariates present within the year preceding the index date of LBP diagnosis could be factored into PSM. Covariates were specified a priori based on their association with constipation as described in a literature review of common causes of constipation.^9^ These included medications used for LSR and endocrine, neurologic, and opioid use disorders. Irritable bowel syndrome was propensity matched to improve the validity of results because IBS can cause diarrhea or constipation.^31^

Neurological disorders were matched by choosing diagnoses within the ICD-10 category G00-G99 with the greatest between-cohort baseline difference. This was intentional because controlling for all neurological conditions (eg, neuralgia) could unintentionally exclude patients with LSR. Conversely, the entire ICD-10 category “endocrine, nutritional, and metabolic diseases” (E00-E89) was controlled for as many of these disorders can cause constipation, such as hypothyroidism and diabetes.^9^

Central nervous system medications as listed in the VANDF class CN100^20^ were factored into PSM. These included but were not limited to analgesics, anesthetics, sedatives or hypnotics, anticonvulsants, antidepressants, antipsychotics, lithium salts, and central nervous system stimulants (Supplementary material, Table 5, available at http://links.lww.com/PR9/A128). The drug class for musculoskeletal medications (MS000) was likewise added to PSM. We did not control for corticosteroid medications, which have less evidence of an association with constipation.

### 2.7. Study size

A sample size of 988 was calculated using G*Power and z-tests for logistic regression, with a power of 0.95, α error of 0.05, and assuming a normal distribution. A value of 0.9 was used for R2 to estimate a high level of interaction between covariates: the probability of constipation given the null hypothesis was 0.27, the higher estimate of the prevalence of constipation in adults from a previous review,^28^ and the probability of constipation given the alternative hypothesis was 0.45, the incidence of constipation in patients with sciatica in a previous study.^1^

### 2.8. Statistical methods

TriNetX uses logistic regression to calculate propensity scores for patients in each cohort, and a matching ratio of 1:1 using greedy nearest neighbor matching, with a caliper of 0.01 pooled standard deviations. Baseline characteristics were compared using an independent samples t test for continuous variables and a Pearson χ^2^ test for categorical variables (presented as percentages and frequencies, Table 1). Missing data were examined by comparing the average facts per patient because a similar data density between cohorts suggests a minimal effect of documentation bias.^35^

**Table 1:** Baseline characteristics.

Characteristic	Before matching			After matching		
	LSR	Nonradicular LBP	*P*	LSR	Nonradicular LBP	*P*
N	503,760	1,071,498		503,062	503,062	
Age	36.9 ± 8.22	33.9 ± 9.02	<**0.001**	36.9 ± 8.21	37 ± 8.25	**0.0139**
Sex						
Female	288,696 (57.31%)	622,506 (58.10%)	<**0.001**	288,269 (57.30%)	288,104 (57.27%)	0.7394
Male	214,918 (42.66%)	448,640 (41.87%)	<**0.001**	214,648 (42.67%)	214,794 (42.70%)	0.768545
Race						
Black	87,454 (17.36%)	233,811 (21.82%)	<**0.001**	87,452 (17.38%)	85,734 (17.04%)	**<0.001**
White	327,023 (64.92%)	630,010 (58.80%)	<**0.001**	326,365 (64.88%)	328,740 (65.35%)	**<0.001**
Asian	10,972 (2.18%)	27,382 (2.56%)	<**0.001**	10,971 (2.18%)	10,598 (2.11%)	**0.0102**
American Indian	2,350 (0.47%)	4,657 (0.44%)	**0.0050**	2,347 (0.47%)	2,171 (0.43%)	**0.0086**
Pacific Islander	895 (0.18%)	1,916 (0.18%)	0.8731	894 (0.18%)	836 (0.17%)	0.1628
Ethnicity						
Hispanic/Latino	41,904 (8.32%)	102,477 (9.56%)	**<0.001**	41,895 (8.33%)	41,250 (8.20%)	**0.0195**
Not Hispanic/Latino	279,116 (55.41%)	573,973 (53.57%)	**<0.001**	278,619 (55.39%)	280,955 (55.85%)	**<0.001**
Conditions (ICD-10)						
Cerebral infarction (I63)	1,564 (0.31%)	2,763 (0.26%)	**<0.001**	1,562 (0.31%)	1,306 (0.26%)	**<0.001**
Episodic and paroxysmal disorders (G40-G47)	92,053 (18.27%)	163,633 (15.27%)	**<0.001**	91,725 (18.23%)	91,015 (18.09%)	0.0663
Multiple sclerosis (G35)	2,191 (0.44%)	3,345 (0.31%)	**<0.001**	2,177 (0.43%)	1,889 (0.38%)	**<0.001**
Myopathy (G72.9)	323 (0.06%)	482 (0.05%)	**<0.001**	321 (0.06%)	246 (0.05%)	**0.0016**
Cerebral palsy (G80)	454 (0.09%)	1,422 (0.13%)	**<0.001**	454 (0.09%)	284 (0.06%)	**<0.001**
Irritable bowel syndrome (K58)	6,718 (1.33%)	9,988 (0.93%)	**<0.001**	6,654 (1.32%)	6045 (1.20%)	**< 0.001**
Endocrine, nutritional, and metabolic diseases (E00-E89)	131,502 (26.10%)	231,202 (21.58%)	**<0.001**	131,072 (26.06%)	130,497 (25.94%)	0.1912
Autoimmune thyroiditis (E06.3)	1,947 (0.39%)	3,265 (0.31%)	**<0.001**	1,937 (0.39%)	1,712 (0.34%)	**<0.001**
Thyrotoxicosis (E05.0)	1,290 (0.26%)	2,248 (0.21%)	**<0.001**	1,285 (0.26%)	1,081 (0.22%)	**<0.001**
Cushing syndrome (E24)	201 (0.04%)	334 (0.03%)	**0.0055**	201 (0.04%)	175 (0.04%)	0.1798
Opioid-related disorders (F11)	6,464 (1.28%)	11,956 (1.12%)	**<0.001**	6,449 (1.28%)	5,905 (1.17%)	**<0.001**
Medications (VANDF)						
CNS medications (CN000)	248,421 (49.31%)	454,121 (42.38%)	**<0.001**	247,723 (49.24%)	246,508 (49.00%)	**0.0153**
Opioid analgesics (CN101)	170,884 (33.92%)	293,119 (27.36%)	**<0.001**	170,272 (33.85%)	169,086 (33.61%)	**0.0123**
Nonopioid analgesics (CN103)	157,423 (31.25%)	283,628 (26.47%)	**<0.001**	156,958 (31.20%)	156,009 (31.01%)	**0.0409**
Nonsteroidal anti-inflammatory analgesics (CN104)	106,775 (21.20%)	207,688 (19.38%)	**<0.001**	106,629 (21.20%)	105,570 (20.99%)	**0.0096**
Sedatives/hypnotics (CN300)	98,183 (19.49%)	155,912 (14.55%)	**<0.001**	97,729 (19.43%)	97,242 (19.33%)	0.2193
Anticonvulsants (CN400)	56,364 (11.19%)	69,959 (6.53%)	**<0.001**	55,679 (11.07%)	51,913 (10.32%)	**<0.001**
Musculoskeletal medications (MS000)	201,907 (40.08%)	346,234 (32.31%)	**<0.001**	201,211 (40.00%)	199,930 (39.74%)	**0.0091**

*P* values < 0.05 in bold.

CNS, central nervous system, LBP, low back pain, LSR, lumbosacral radiculopathy, VANDF, Veterans Health Administration National Drug File.

## 3. Results

### 3.1. Participants

A large sample size was identified for each cohort. Before PSM, there were 503,760 patients in the LSR cohort and 1,071,498 in the nonradicular LBP cohort. After PSM, there were 503,062 patients in each cohort. The LSR cohort had a significantly greater age, a greater incidence of most comorbidities except for muscular dystrophy and IBS, and greater utilization of medications (Table 1).

To safeguard PHI and improve performance times, TriNetX obfuscates patient counts within their “Explore Cohort tool,” in which results are limited to ∼10,000 patients per HCO. Analyses of baseline characteristics and outcomes include all patients meeting the study selection criteria. In accordance with the limitation of exploring the entire cohorts in greater detail, an analysis of 358,287 of patients randomly selected from the LSR cohort was performed to characterize the frequency of included diagnoses. The most common diagnoses in the LSR cohort were radiculopathy in the lumbar region (ICD-10: M54.16), which was present in 45% of patients, followed by sciatica (ICD-10: M54.3, 38%), lumbago with sciatica (ICD-10: M54.4, 37%), and radiculopathy in the lumbosacral region (ICD-10: M54.17, 27%). Lumbosacral root disorders, not elsewhere classified (ICD-10: G54.4) and radiculopathy, sacral and sacrococcygeal region (ICD-10: M54.18) were uncommon and found in <1% of the LSR cohort. The distribution of diagnoses suggested a successful identification of the target population for our LSR cohort.

### 3.2. Descriptive data

The number of data points between cohorts were compared, showing a high average number of facts per patient (LSR 812 and nonradicular 698), which was similar between cohorts, suggesting a minimal effect of documentation bias or missing information. Because of the large sample size and large prematching differences, there were statistically significant differences in certain variables postmatching. A visual diagnostic showed that the propensity scores between cohorts were well-balanced despite these differences (Supplementary material, Figure 2, available at http://links.lww.com/PR9/A128).

### 3.3. Key results

The odds of constipation were equivalent between the LSR and nonradicular LBP cohorts (LSR: 10.8%, nonradicular LBP: 10.9%; odds ratio [OR] [confidence interval] = 0.99 [0.98-1.0], *P* = 0.251). The odds remained nearly identical after propensity score matched analysis (LSR: 10.8%, nonradicular LBP: 11.1%; OR [confidence interval] = 0.98 [0.97-0.99], *P* = 0.003). Although the *P* value after PSM indicated statistical significance of the OR, the magnitude of the OR remained nearly equal to the null value of 1.0.

### 3.4. Sensitivity analysis

Although sensitivity analysis was planned for this study using the E-value,^44^ this was not applicable because of the lack of an identified increase in odds of constipation in the LSR cohort.

## 4. Discussion

This purpose of this retrospective study was to investigate the possible association between LSR and constipation using a real-world national database, with the hypothesis that adults with LSR have increased odds of developing constipation compared with those with nonradicular LBP. To the best of our knowledge, this study was the largest of its kind including 503,062 patients in each LSR and nonradicular LBP cohorts using the large TriNetX platform.

Initial analyses indicated that patients with LSR had equivalent odds of developing constipation than nonradicular LBP before PSM (OR 0.99), and this was unchanged (OR 0.98) after matching for demographics, comorbidities, and medications. This suggests that there is no clinically significant association between LSR and constipation compared with nonradicular LBP.

This study does not rule out an association between constipation and LBP in general, given that about 11% of patients in each cohort developed constipation within the year after diagnosis. Although this rate could be reflective of the high prevalence of constipation in the general population,^28^ our study only analyzed incident cases of constipation over one year. The percentage of patients developing constipation in both LSR and nonradicular LBP cohorts could be congruent with previous research that identified an association between LBP and constipation.^33,49^

The lack of an independent association between LSR and constipation suggests that the spinal defecatory reflex is not diminished to a significant degree or is not required to maintain colonic motility even if abolished in these patients. It is possible that the remaining functional lumbosacral roots permit continued function of this reflex or that the enteric nervous system maintains colonic motility despite the loss of the spinal defecatory reflex.

The results of this study should be contrasted with previous studies reporting an association between LBP and urinary incontinence.^16,51^ Although patients with LSR are more at risk of urinary incontinence,^16^ our study shows this relationship does not hold true for constipation. It is possible that there is a lower threshold to urinary incontinence in LSR, whereas bowel dysfunction is only affected in those with more severe forms of LSR such as CES. This explanation relates to neurophysiological differences because the bladder seems to be more reliant on its spinal innervation as compared to the colon.

Given that constipation may develop subsequent to either LSR or nonradicular LBP diagnoses with similar frequency, clinicians should be mindful of the constipating effects of medications used to treat pain in patients with LBP such as opioids, sedatives, and anticonvulsants. When treating LBP, clinicians may prescribe medications with a reduced risk of constipation, or over a shorter duration, or recommend evidence-based nonpharmacologic therapies.

One previous study found that increasing pain severity is positively associated with constipation severity.^2^ This may explain the equivalent rate of constipation in both LSR and nonradicular LBP cohorts, given both conditions are inherently painful. This previously identified association underscores the importance of treating pain in patients with low back conditions with nonpharmacologic interventions rather than opioid medications, which can increase the risk of constipation.^22^ Indeed, a recent clinical practice guideline from the American College of Physicians provided a strong recommendation for superficial heat, massage, acupuncture, or spinal manipulation for acute or subacute LBP.^25^

Strategies to promote movement and exercise could benefit patients with LBP and constipation because physical inactivity is associated with both conditions. Although the aforementioned treatments for pain may enable patients to be more active, it may be beneficial to directly encourage evidence-based exercise therapies for LBP, such as tai chi or yoga, or refer patients for physical therapy.^25^ In general, lifestyle factors such as regular walking or exercise are protective against LBP.^30^

Constipation in those with LBP is an important interdisciplinary consideration. Providers treating the spine should communicate with primary care or gastroenterology providers to facilitate an optimal outcome for their patients because constipation has unwanted sequelae such as hemorrhoids and diverticular disease. In exchange, management of constipation may reduce LBP-related exacerbations because straining, eg, during elimination, often exacerbates discogenic LSR.^38^

## 5. Limitations

Although we accounted for differences between cohorts using PSM, there are numerous residual confounding variables such as those related to pain severity,^2^ physical activity level,^53^ socioeconomic status, stress, hormonal changes, water intake,^53^ and dietary variables, which were not available in our data set. Another potential confounder is pelvic floor dysfunction, which has a limited association to LBP but strong association with constipation.^9^ However, this diagnosis is poorly represented in the ICD coding system, making it difficult to account for in our study.

We were unable to determine the severity of constipation in each cohort. Data from a patient-reported outcome index for constipation such as the Constipation Scoring System was unavailable in the TriNetX platform, and if present, could have enabled this information to be known. Although the incidence of constipation was similar between cohorts, it is possible that the severity of constipation differed. In addition, data regarding the dose and duration of laxative medications were unavailable in TriNetX. As laxatives were part of the composite outcome for constipation, this missing information could have affected study results.

Some patients could have been misclassified in the EHR. This could happen if patients received an incorrect diagnosis or older, inactive comorbidities were copied into the current chart. Prevalent users could have been included if they received care outside of the HCOs included in TriNetX and had a previous history of constipation and LBP. In addition, some patients could have been lost to follow-up. Although there is no guarantee that all included patients had incident LBP, a washout period to exclude previous LBP minimized this bias by including patients at a similar time point coinciding with the index date of LBP diagnosis.

We were unable to determine the severity of LSR. Patient-reported outcome assessments, imaging results, and pain scores are unavailable in TriNetX. Although we attempted to create a uniform cohort of patients with LSR by excluding those with CES, the remaining patients could have differed with regards to having mild, moderate, or severe nerve root compression and corresponding neurologic deficits.

Patients with LSS were excluded from this study. Although LSS commonly causes LSR, it may have significant clinical and pathophysiological differences from discogenic LSR. As the onset of neurologic deficits is more insidious, a longer follow-up may be required. In addition, comorbidities such as dementia would need to be considered within the study design. This study could be repeated with a focus on patients age 50 and older with LSS. Finally, our study design did not permit us to investigate constipation as a result of CES, in which lumbosacral nerve roots are more severely injured. Most patients with LSR have an injury to one or 2 nerve roots, usually L5 or S1,^21^ whereas those with CES have multiple nerve roots injured and are more likely to have involvement of the sacral roots, which are important for bowel and bladder function.^41^ Further research could compare constipation in patients with LSR to those with CES.

## 6. Conclusions

The results of this study refute the hypothesis that patients with LSR have increased odds of constipation compared with those with nonradicular LBP, given that the results showed equivalent odds of this outcome using a large propensity-matched sample. Although clinicians should be vigilant to recognize constipation in patients with LBP, they should understand that LSR is unlikely to be directly causative. Numerous variables contribute to constipation which may be present in LBP in general, including increasing pain severity, physical inactivity, and medication side effects. In addition, future researchers should consider using TriNetX so that real-world EHR data can inform clinical practice.

## Appendix A. Supplemental digital content

Supplemental digital content associated with this article can be found online at http://links.lww.com/PR9/A128.

## Supplementary Material

SUPPLEMENTARY MATERIAL
