# Supplementary material for: Association between radicular low back pain and constipation: a retrospective cohort study using a real-world national database
Source: Pain Rep. 2021 Aug 26;6(3):e954. doi: 10.1097/PR9.0000000000000954 (PMC8397289; doi:10.1097/PR9.0000000000000954)
Supplement: SUPPLEMENTARY MATERIAL [file painreports-6-e954-s001.pdf]

Supplementary material

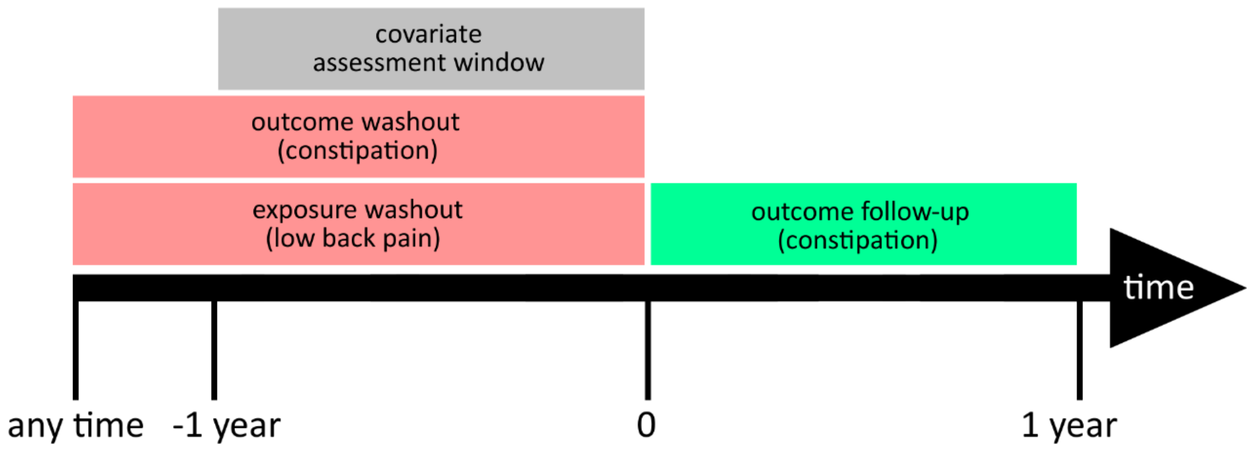

Figure 1: Study design and covariate assessment window, washout periods, and follow-up period

Table 1: Exclusions for both cohorts

Rationale	ICD-9 Code	ICD-9 Definition
Alternate neurological injury	353.1	Lumbosacral plexus lesions
	336.8	Other myelopathy
	336.9	Unspecified disease of spinal cord
	724.0	Spinal stenosis other than cervical
Serious pathology	324.1	Intraspinal abscess
	344.6	Cauda equina syndrome
	730	Osteomyelitis
	733.13	Pathologic fracture of vertebrae
	805	Fracture of vertebral column without mention of spinal cord injury
	806	Fracture of vertebral column with spinal cord injury
	C00-C96	Malignant neoplasm
Noninfective enteritis and colitis	K50	Crohn's disease [regional enteritis]
	K51	Ulcerative colitis
	K52	Other and unspecified noninfective gastroenteritis and colitis

Table 2: Radicular low back pain inclusion codes with Boolean "OR"

ICD-10 Code(s)	ICD-9 Definition
G54.4	Lumbosacral root disorders, not elsewhere classified
M54.3	Sciatica
M54.4	Lumbago with sciatica
M54.16	Radiculopathy, lumbar region
M54.17	Radiculopathy, lumbosacral region
M54.18	Radiculopathy, sacral and sacrococcygeal region

Table 3: Additional exclusions for non-radicular low back pain

ICD-9 Code	Definition
722.1	Displacement of thoracic or lumbar intervertebral disc without myelopathy
722.73	Intervertebral disc disorders with myelopathy, lumbar region
722.93	Other and unspecified disc disorder, lumbar region
724.3	Sciatica
724.4	Thoracic or lumbosacral neuritis or radiculitis, unspecified
756.11	Spondylolysis, lumbosacral region
756.12	Spondylolisthesis
353.4	Lumbosacral root disorders, not elsewhere classified
729.2	Neuralgia and neuritis, unspecified
724.03	Spinal stenosis, lumbar region with neurogenic claudication
724.02	Spinal stenosis, lumbar region without neurogenic claudication

Table 4: Outcome of interest phenotype for constipation with Boolean "OR"

Code	Definition
International Classification of Diseases ICD-9	
546.00	Constipation, unspecified
546.09	Chronic idiopathic constipation
546.01	Slow transit constipation
546.02	Outlet dysfunction constipation
546.09	Other constipation
VANDF GA200, Veterans Health Administration National Drug File: Laxatives	
GA201	Bulk-forming Laxatives
GA202	Hyperosmotic Laxatives
GA203	Lubricant Laxatives
GA204	Stimulant Laxatives
GA205	Stool Softener
GA206	Carbon Dioxide-releasing Laxatives
GA209	Laxatives, Other

Table 5: Medications factored into propensity score matching

**Veterans Health Administration National Drug File drug classes**	**Examples (not comprehensive)**
Opioid Analgesics (CN101)	Hydrocodone, oxycodone, meperidine, hydromorphone, morphine
Sedatives/Hypnotics (CN302)	Benzodiazepines: Diazepam, alprazolam, lorazepam, clonazepam
Anticonvulsants (CN400)	Oxcarbazepine, lamotrigine, gabapentin, carbamazepine, topiramate
Non-Steroidal Anti-Inflammatory Analgesics (CN104)	Ibuprofen, naproxen
Non-opioid analgesics (CN103)	Acetaminophen, aspirin
Others considered within CN100	Pregabalin, amitriptyline
Musculoskeletal medications (MS000)	Skeletal muscle relaxants: Cyclobenzaprine, methocarbamol

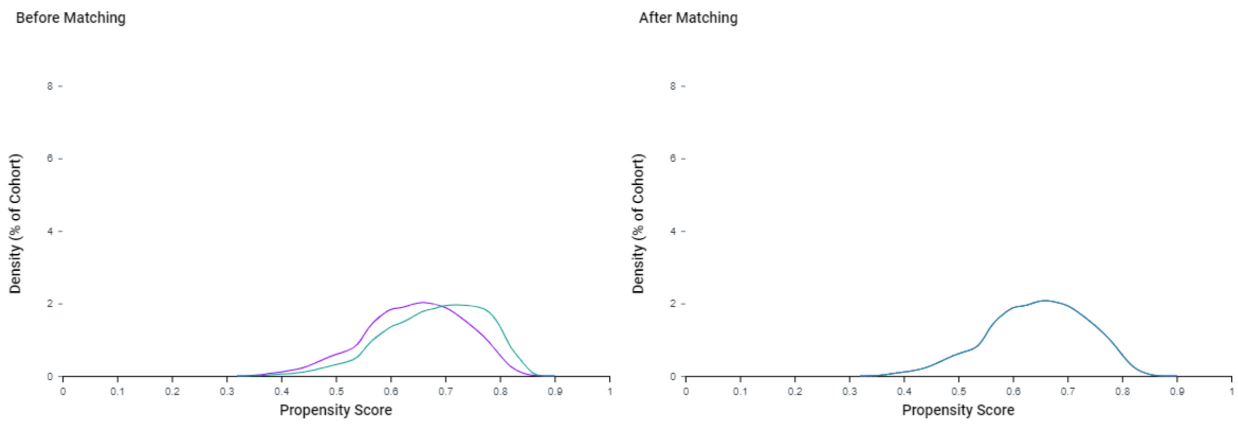

Figure 2: Propensity scores before and after matching. Purple-colored line represents lumbosacral radiculopathy cohort while green represents non-radicular low back pain cohort.
